# The Effect of Isotropic One-Process and Two-Process Surface Textures on the Contact of Flat Surfaces

**DOI:** 10.3390/ma12244092

**Published:** 2019-12-07

**Authors:** Pawel Pawlus, Wieslaw Zelasko, Andrzej Dzierwa

**Affiliations:** 1 Faculty of Mechanical Engineering and Aeronautics, Rzeszow University of Technology, Powstancow Warszawy 8 Street, 35-959 Rzeszow, Poland; adzierwa@prz.edu.pl; 2 Faculty of Mechanics and Technology, Rzeszow University of Technology, Kwiatkowskiego Street, 37-450 Stalowa Wola, Poland; w.zelasko@prz.edu.pl

**Keywords:** surface topography, two-process surfaces

## Abstract

The contact of random modeled one- and two-process textures with smooth, flat surfaces is discussed in this paper. An elastic-plastic contact model was applied, assuming a distributed radius of summits. A one-process surface was characterized by the standard deviations of height and the correlation length; however, it also had a two-process texture by the standard deviations of the plateau and valley structures, the material ratio at the transition point, and the correlation lengths of the plateau and valley parts. It was found that the contact characteristics depended on the height and spatial properties of the surface texture. The plateau part governs the contact characteristics of two-process surfaces, while the effect of the valley surface portion is smaller. The plastic deformation leads to a smaller effect of the surface texture on the contact characteristics.

## 1. Introduction

A correct characterization of a contact between rough surfaces is important for analyzing many tribological problems. Several probabilistic contact models were proposed. The basic elastic contact model was developed by Greenwood and Williamson [1]. The rough surface is represented in this model by a set of summits of the same radius of curvature and Gaussian distribution of their heights. Elastic–plastic statistical contact models were developed on the basis of theoretical consideration [2,3] or the finite elements method (FEM) [4,5,6,7]. In these approaches, the single asperity contact models are incorporated into rough surface contact [8]. It is believed that numerical methods [9,10] are better than statistical ones for modeling the contact between rough surfaces. FEM can be also used for simulating the contact between two rough surfaces [11,12].

The surface topography effect on the contact between rough surfaces was analyzed using a plasticity index [1]

(1) $$\Psi =\frac{E^{\prime }}{H}{\left(\frac{\sigma_{s}}{R}\right)}^{1/2},$$

where H—hardness of the softer material, E′—Hertz elastic modulus, *σ_s_*—standard deviation of asperity height, and R—mean radius of summits curvature. 

The contact is elastic for plasticity index values smaller than 0.6 and plastic for plasticity index values larger than 1. When the plasticity index is higher, the surface is more inclined to plastic deformation (and wear). During the analysis of the contact between rough surfaces, not only height but also horizontal parameters should be taken into consideration. From among them, a correlation length is commonly used. It is a distance at which the autocorrelation function slowly decays to a given value (usually 0.1 [13]). It was found [14] that a decrease in the roughness height led to the elastic deformation, but an increase in the correlation length led to a decrease in the maximum contact pressure. It was found [15] that an increase in the plasticity index due to the change in the surface topography of Gaussian ordinate distribution led to increases in the contact area and the contact load for the same separation. The authors of paper [16] analyzed the effect of the correlation length on the real area of contact. It was found that a higher correlation length corresponded to a higher number of contact points and therefore a larger contact area for the same separation based on surface heights.

Typically, the contact of surfaces of Gaussian ordinate distribution was analyzed. In Reference [17], the contact of surfaces of different ordinate distribution (the skewness Ssk was in the range from −2 to +2) was studied. It was found that skewness affected the contact load and the contact area. A separation based on asperity heights for the same load or the same contact area is lower for more negative skewness for the same standard deviation of surface height. For surfaces of asymmetric ordinate distribution, similar to Gaussian topographies, the contact load is proportional to the contact area, except for very large loads.

Jang and Peng [18] found that the contact of anisotropic surfaces of negative skewness was more elastic than that of surfaces of Gaussian ordinate distribution and the same standard deviation of surface height. A similar tendency was confirmed for isotropic surfaces, considering the asperities’ interaction [19].

The obtained findings were confirmed experimentally only for the effect of surface height on the rough surface contact; when the roughness height was higher, the deformations were bigger [20,21].

The functional properties of two-process surfaces can be better than those of one-process textures [22,23,24,25]. Two-process surfaces can be machined or formed during wear. Only a few publications about the contact of two-process surfaces have been presented so far [26,27,28]. Greenwood and Williamson [1] thought that two-process worn surfaces were characterized by the Gaussian distribution of heights of summits from the plateau surface part. Leefe [26] believed that the contact of two-process surfaces was governed by the Gaussian distribution of the asperity heights from the plateau surface portion. This finding was partially confirmed during experimental investigations [29].

The aim of this work is to study the effect of isotropic one-process and two-process surface textures on the contact of flat surfaces. Not only height but also horizontal parameters will be taken into consideration.

## 2. Surface Topography Modeling

Modeled isotropic one- and two-process surfaces were analyzed. Each isotropic texture was characterized by the standard deviation of surface heights and the correlation length, which was the same in two orthogonal directions. Isotropic surfaces of Gaussian ordinate distribution were modeled using the procedure developed by Wu [30]. This method was selected because it better modeled the surface topographies of comparatively large correlation lengths than the other existing approaches [31].

**Figure 1:**
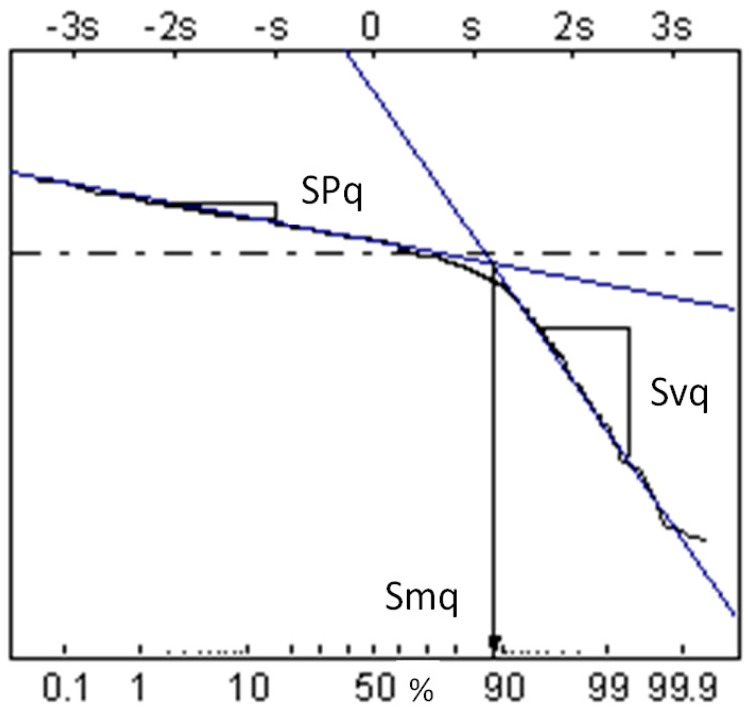
Material probability curve of two-process surface.

**Figure 2:**
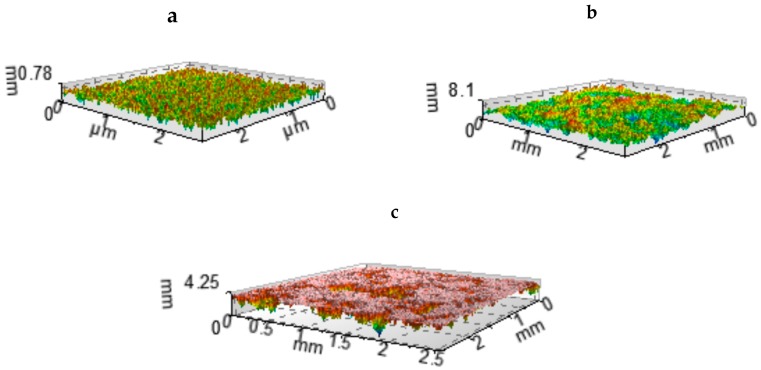
Example of two-process surface modeling: the plateau surface: Sq = 0.1 µm, CL = 50 µm (**a**), the valley surface: Sq = 1 µm, CL = 250 µm (**b**), two-process surface: Spq = 0.1 µm, Svq = 1 µm, and Smq = 84.13% (**c**). Sq: standard deviation of height, CL: correlation length, Spq: standard deviation of the plateau part, Svq: standard deviation of the valley part, Smq: material ratio at plateau-to-valley transition.

Two-process surfaces were modeled by the imposition of the Gaussian one-process plateau surface on the one-process valley Gaussian surface [31]. The description of the two-process surface is based on the material probability curve (cumulative distribution of surface heights) in which the material ratio is expressed as a Gaussian probability in standard deviation values (−3s = 0.13%, −2s = 2.28%, −s = 15.8%, 0 = 50%, s = 84.13%, 2s = 97.72%, 3s = 99.87%). For the random two-process surface, the material probability curve has two linear regions. The standard deviation of the plateau structure (the Spq parameter) is the slope of the linear regression through the plateau part, while the standard deviation of the valley structure (the Svq parameter) is the slope of the linear regression through the valley portion. The intersection point of abscissa Smq expresses the separation between plateau and valley textures [31,32]. Figure 1 presents an example of the material probability curve of the two-process texture. These textures were characterized by the following parameters: Spq, Svq, Smq, the correlation length of the plateau surface, CLp, and the correlation length of the valley surface, CLv. Figure 2 shows an example of two-process surface modeling.

Each modeled surface (one- and two-process) consisted of 512 × 512 points. The sampling interval in the perpendicular direction was set to 5 µm.

## 3. Calculation of Contact Characteristics

**Figure 3:**
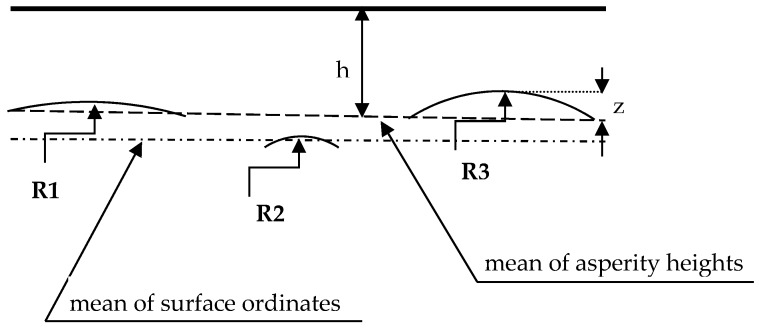
A scheme of the contact of two rough surfaces, h—the separation based on surface heights, z—the height of summit, **R1**, **R2**, **R3** = radii of individual summits.

In the contact calculation of one- and two-process surfaces, the following fundamental assumptions [1] were adopted: asperities are spherical near their summits, there is no interaction between summits, and only the asperities are deformed. A point on a surface was chosen to be a summit if its ordinate was higher than those of eight neighboring points. The radius of each peak was calculated as reciprocal of its average curvature in perpendicular directions. The summit curvature was computed using a three-point formula [33]. For each summit that is in contact with the interference, the contact area and the contact load were determined using a JG (Jackson and Green) elastic–plastic contact [6] model. The total contact area A and the total contact load P were calculated for various separations h (see Figure 3) based on surface heights [4]. The vertical range of summits ordinates was divided into many intervals. The total contact load P and the total contact area A were computed by summing the contributions of all the contacting asperities located above a given separation. In calculation, each summit had its own radius of curvature. Similar procedures were used in Reference [33] for the CEB (Chang, Etsion and Bogy) model [2], in Reference [34] and [35] for the GW (Greenwood and Williamson) model [1], as well as in Reference [36] for the JG model [2].

The contact of steel-on-steel flat surfaces was considered with composite Young modulus E’ = 113.115 GPa. It was assumed that the rough disc with 4.3 GPa hardness contacted the smooth flat surface.

## 4. Results and Discussion

### 4.1. Contact of One-Process Surfaces

Six modeled surfaces were analyzed. Smooth textures I5001, I10001, and I25001 had the same standard deviation of roughness height, Sq = 0.1 µm. However, the correlation length (CL) increased from 50 µm (surface I5001), through 100 µm (I10001), to 250 µm (I25001). Rough surfaces I5005, I10005, and I25005 had spatial parameters identical to surfaces I5001, I10001, and I25001 respectively, but the roughness height described by the Sq parameter increased from 0.1 µm to 0.5 µm. In Table 1, the standard deviation of roughness height *σ_s_*, the summit density Sds, and the mean radius of summit curvature R are presented [33]. One can see that an increase in the correlation length CL led to increases in *σ_s_* and R as well as to a decrease in Sds. Due to increases in roughness height parameters, *σ_s_* increased, while the mean radius of curvature decreased. In Table 1, the plasticity index Ψ is also included. Due to an increase in the correlation length, the plasticity index decreased. The plasticity index increased owing to the growth of the surface height.

**Figure 4:**
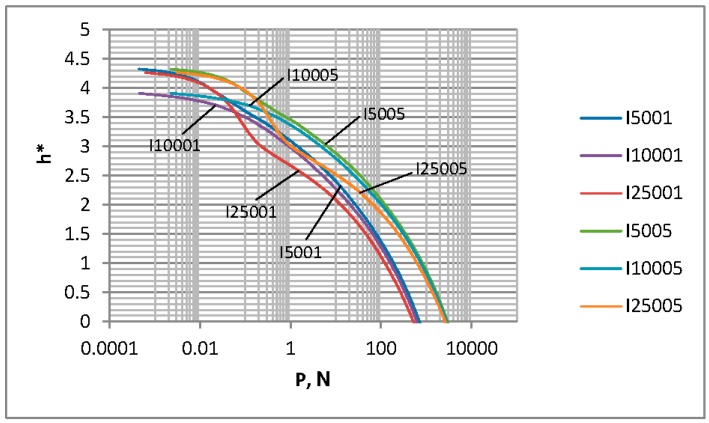
The dimensionless separation versus the load for random one-process isotropic surfaces.

**Figure 5:**
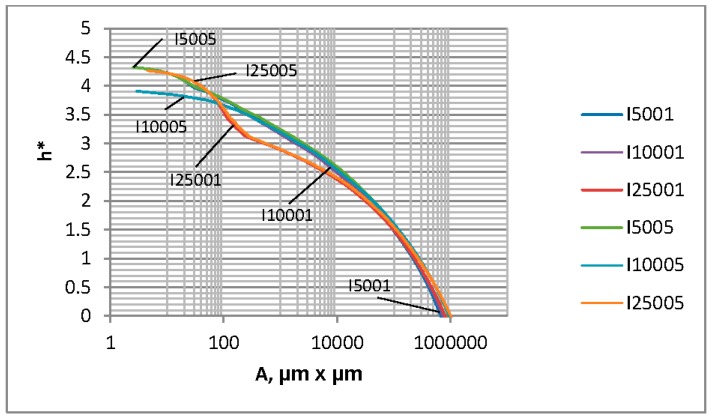
The dimensionless separation versus the contact area for random one-process isotropic surfaces.

**Figure 6:**
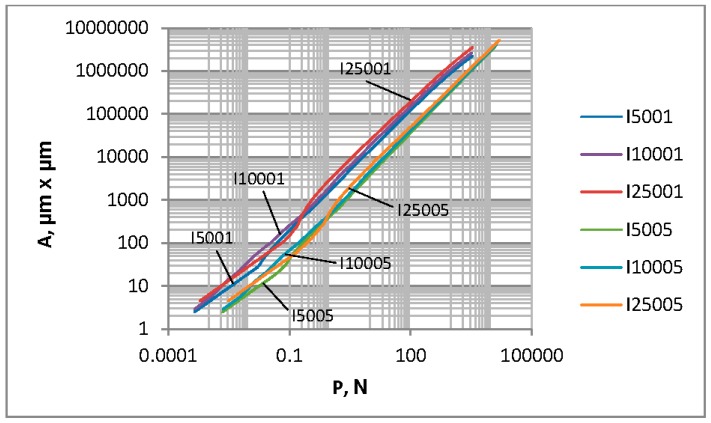
The contact area versus the load for random one-process isotropic surfaces.

Figure 4, Figure 5 and Figure 6 show the contact characteristics of one-process surfaces. For comparison, instead of the separation based on surface heights h, a dimensionless separation h*, which is the ratio of the separation h to the standard deviation of surface heights, was analyzed (similar to many publications, such as Reference [2]). The separation h* versus the load P and the contact area A for various one-process textures are shown in Figure 4 and Figure 5, respectively. Figure 6 presents the contact area A versus the load P.

It is evident from the analysis of Figure 4 that for loads larger than 1 N, the dimensionless separation is higher when the plasticity index is higher. A similar dependence was observed in References [2,3] after the use of other contact models. However, Chang et al. [2] and Zhao et al. [3] analyzed the effect of only roughness height. In the present work, the main wavelength (correlation length) also varied. An increase in the main wavelength for the same roughness amplitude led to a decrease in the dimensionless separation for the same load (Peng at al. [15] obtained similar results); the differences are larger for lower topography height. For larger loads than 100 N, the effect of the roughness height on the dimensionless separation is larger compared to the effect of the main wavelength.

It seems from the analysis of Figure 5 that the effect of surface topography on h* versus A dependence is lower than that on h* versus P relation. The effect of surface texture on the separation between contacting rough surfaces for a given contact area is dependent on this area. When A is between 300 and 10,000 µm^2^, the h* versus P relation depends on the correlation length, not the roughness height; the dimensionless separation is smaller for the larger main wavelength. For a contact area larger than 100,000 µm^2^, the separation h* is higher for the bigger roughness amplitude, while the effect of the correlation length on the h* versus P relation is lower.

**Figure 7:**
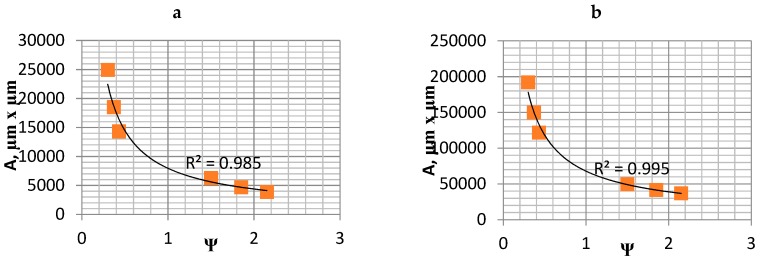
Dependence between the plasticity index of one-process surfaces and the contact area for the load of 10 N (**a**) and 100 N (**b**).

Since the effect of the contact area on the dimensionless separation marginally depends on the surface texture, the dependence shown in Figure 6 is primarily the consequence of that presented in Figure 4. For loads higher than 1 N, the contact area for the same load is higher when the plasticity index is smaller. Similar results were presented in References [2,3]. The effect of the lower plasticity index (corresponding to the elastic contact) is larger than that of the higher one (corresponding to the plastic contact)—see Figure 7. For smaller loads than 1 N, the contact area is larger for smaller roughness heights, and the effect of the correlation length is small.

**Figure 8:**
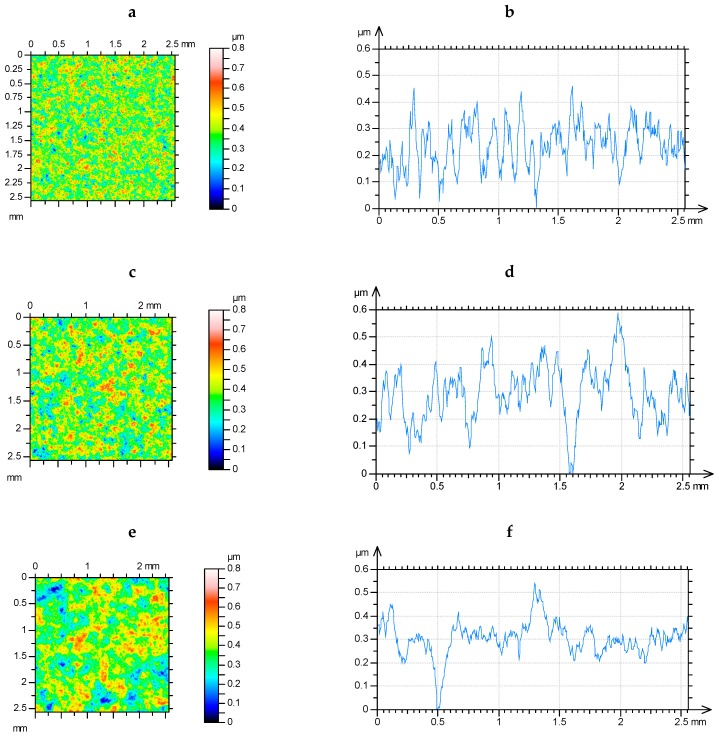
Contour plots (**a**,**c**,**e**) and profiles (**b**,**d**,**f**) of the surfaces I1001 (**a**,**b**), I2001 (**c**,**d**), and I5001 (**e**,**f**).

It is evident after the analysis presented above as well as analyses of other simulated surfaces that the contact characteristics of textures of Gaussian ordinate distribution depend not only on amplitude but also on spatial surface properties. The contour plots and profiles of surfaces I1001, I2001, and I5001 are shown in Figure 8. These surfaces of similar heights differ only by correlation lengths.

### 4.2. Contact of Two-Process Surfaces

**Figure 9:**
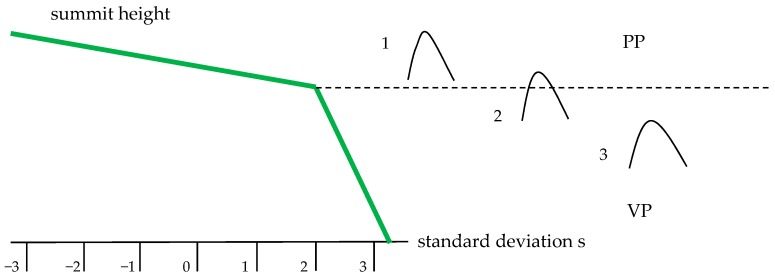
Types of summits on the two-process surface.

During the analysis of contact parameters of two-process surfaces, not all the summits that existed on surface were considered. In order to recognize all the summit types, the cumulative distribution of surface summits should be analyzed (see Figure 9). Similar to the material probability curve (Figure 1), typically there are two parts of the profile obtained from heights of summits—plateau and valley [37]. There are three types of summits of the two-process surface. For a summit of type 1, both the summit highest point and its neighboring points belong to the plateau part (PP). The highest point of summit of type 2 belongs to the plateau part, while the neighboring points to the valley part (VP). A summit of type 3 belongs entirely to the valley part. In the study of contact, only summits of types 1 and 2 should be analyzed. Therefore, the following parameters are important in the contact of two-process surfaces: R2p—the mean radius of summits curvature computed for summits of types 1 and 2, Sds2p—the density of summits of types 1 and 2 and σ2ps—the standard deviation of asperity heights of the two-process surface, which is the slope of the upper straight line visible in Figure 9 [37]. The authors of paper [38] used a similar procedure in order to obtain the contact parameters of two-process surfaces.

Table 2 lists the parameters Spq, Svq, Smq, CLp, CLv, R2p, σ2ps, and Sds2p of analysed two-process surfaces.

All analyzed surfaces were characterized by the same standard deviation of the plateau height Spq; however, the values of the parameters Svq and Smq varied. One can see after a comparison of the parameters of the one-process and two-process surfaces characterized by the same standard deviation of height Sq and the correlation length CL (one-process textures—Table 1) and the standard deviation and the correlation length of the plateau part Spq and CLp, respectively (two-process surfaces—Table 2) that the addition of the valley part caused a decrease in the mean radius of curvature R2p compared to R. There was also a marginal increase in σ2ps compared to *σ_s_*, especially for a smaller correlation length of 50 µm. The standard deviation of asperity height was lower than the standard deviation of surface ordinates; this difference was larger when the correlation length was smaller. A decrease in the radius R2p compared to R was typically larger for the smaller correlation length of the valley part CLv. Changes in R2p compared to R were larger for the higher standard deviation of the valley part Svq. The relative decrease of the mean radius of the summit curvature caused by the valley part presence was larger for the higher correlation length of the plateau part CLp. The ratio of the biggest to the smallest mean radii of curvature of all the analyzed two-process textures was about 2.5. For the same plateau surface part, the summit density of two-process textures is bigger for the valley surface of a smaller correlation length CLv. However, for the same valley surface part, the summits density Sds2p is bigger for the plateau surface of the smaller correlation length CLp. The ratio of the highest to the lowest summit density of all the analyzed two-process textures is about 1.8.

**Figure 10:**
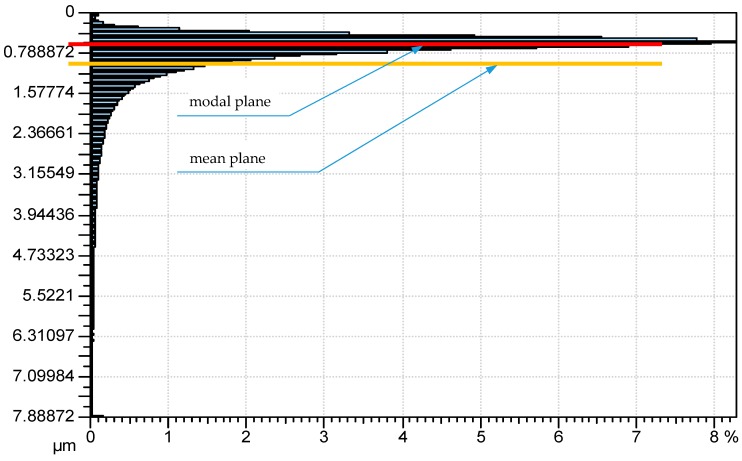
Ordinate distribution of two-process surface.

**Figure 11:**
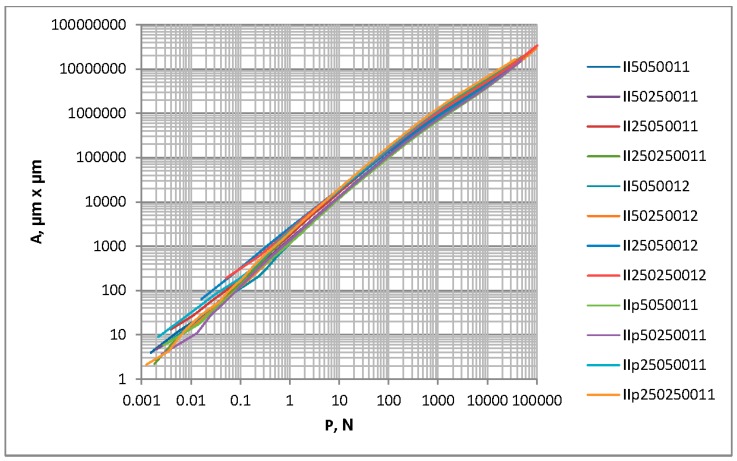
The contact area versus the load for random two-process isotropic surfaces, Spq = 0.1 µm.

**Figure 12:**
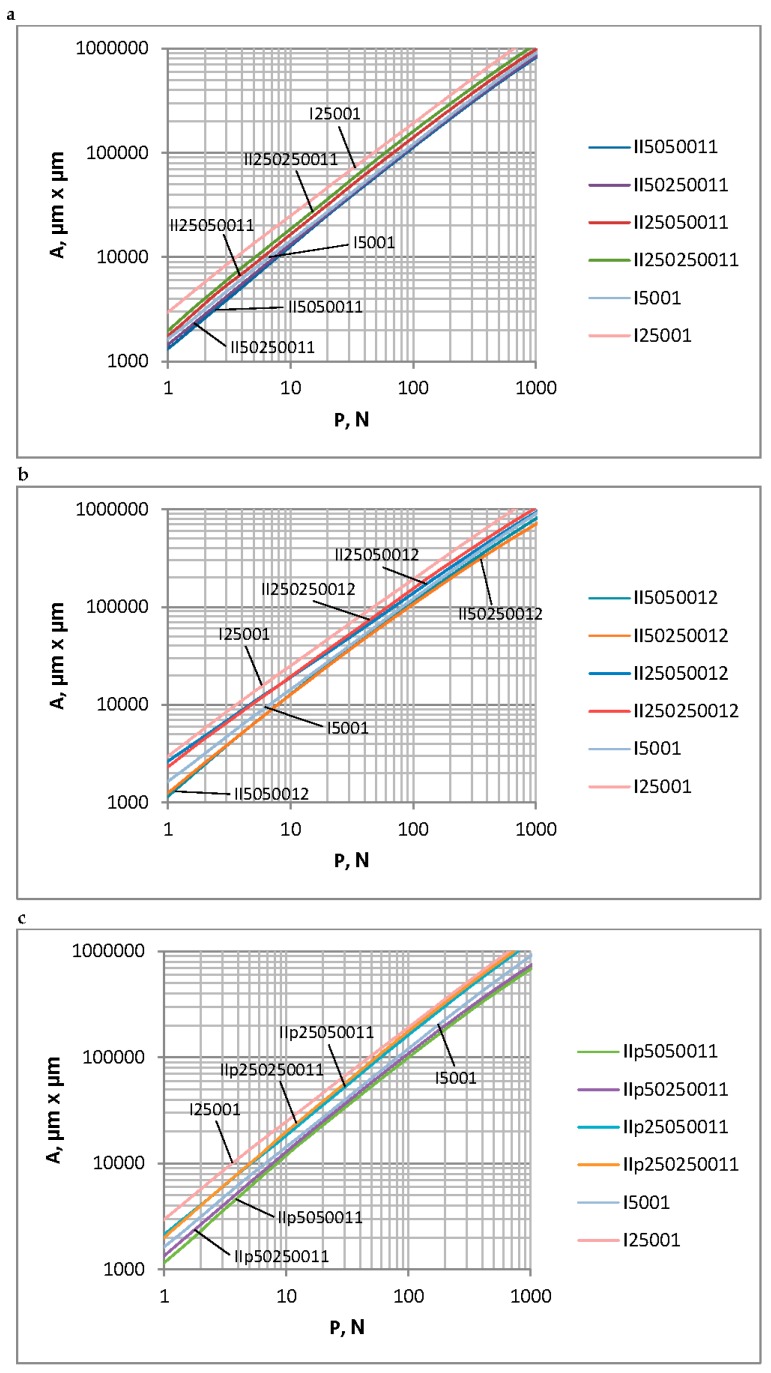
The contact area versus the load for random one-process and two-process isotropic surfaces, characterized by the Spq (Sq) parameter of 0.1 µm. (**a**) two-process surfaces characterized by the transition material ratio of 84% and the Svq parameter of 1 µm and one-process surfaces (**b**) two-process surfaces characterized by the transition material ratio of 84% and the Svq parameter of 2 µm and one-process surfaces (**c**) two-process surfaces characterized by the transition material ratio of 50% and the Svq parameter of 1 µm and one-process surfaces.

There is a problem in correctly determining the reference plane in the contact of two-process surfaces. When the contact of one-process topographies is studied, typically the separation above the mean plane is analyzed. It corresponds to the modal value of heights. However, for two-process surfaces, the mean plane is not the correct reference plane, since the modal plane typically lies above the mean plane. Figure 10 presents the ordinate distribution of a measured two-process surface with modal and mean planes. This difference is larger for a higher Svq/Spq ratio. Therefore, there would be a problem in comparing the separation versus the load and the separation versus the contact area for the contact of various two-process surfaces. So, only the contact area versus the load will be analyzed in this case. Figure 11 presents the contact area versus the load for two-process textures. One can see from the analysis of Figure 11 that the differences between the presented curves are higher for smaller loads than 1 N. However, for larger loads, the deviations are also considerably large. Therefore, more detailed analysis is necessary. Figure 12 shows selected curves from Figure 11 and for comparison, the contact characteristics of one-process surfaces I5001 and I25001 for loads between 1 and 1000 N.

Figure 12a concerns two-process surfaces characterized by the transition material ratio of 84% and the Svq parameter of 1 µm. An increase in the correlation length of the plateau part CLp led to an increase in the contact area for the same load. When the CLp was higher, the contact area was smaller for the higher correlation length of the valley part CLv. However, for smaller CLp values, the contact area for the given load marginally depended on the main wavelength of the valley part. The contact behavior of the two-process textures characterized by the Smq of 84% and the standard deviation of the valley height of 2 µm are presented in Figure 12b. When the correlation length of the plateau part CLp was larger, the contact area for the same load was also larger. The effect of the correlation length of the valley part on the dependence of the load–contact area seems to be small. Figure 12c shows the load–contact area dependence for surfaces characterized by the Smq parameter of 50% and the Svq parameter of 1 µm. Increases in the correlation lengths of both the plateau and valley parts led to a larger contact area for the same load; however, the effect of the plateau part was higher than that of the valley portion. For the analyzed two-process textures, the high contact area for the same load was obtained for surfaces characterized by the higher correlation lengths of both the plateau and valley parts; however, a small contact area was obtained for the given load for surfaces with smaller correlation lengths of both the plateau and valley parts. The contact behavior of one-process surfaces I5001 and I25001 is also presented in Figure 12. The curve corresponded to surface I25001 lay above the other curves; however, the curve that corresponded to surface I5001 was positioned above curves concerning the surfaces of the smaller correlation length of the plateau part CLp. An addition of the valley portion led to the smaller area of contact for the same load. Similar results were obtained in Reference [37].

Table 3 presents the values of the contact area for two-process surfaces for loads of 10 and 100 N. One can see from the analysis in Table 3 that the contact area was high for surfaces characterized by larger correlation lengths of both plateau and valley portions: IIp250250011, II250250011, and II250250012. Surfaces with the smaller correlation lengths of the valley part and of the larger correlation length of the plateau part—IIp25050011, II25050012, and II25050011—also led to the comparatively large contact area. The contact areas of the other surfaces, featuring a smaller correlation length for the plateau part, were smaller. The biggest contact area of the two-process textures was obtained for surface IIp250250011, which was characterized by the higher correlation lengths of both plateau and valley parts, the Smq parameter of 50%, and the Svq parameter of 1 µm. When the load was 10 N, the ratio between the highest and the smallest contact areas was 1.52, while for the larger load of 100 N, this ratio increased to 1.77.

**Figure 13:**
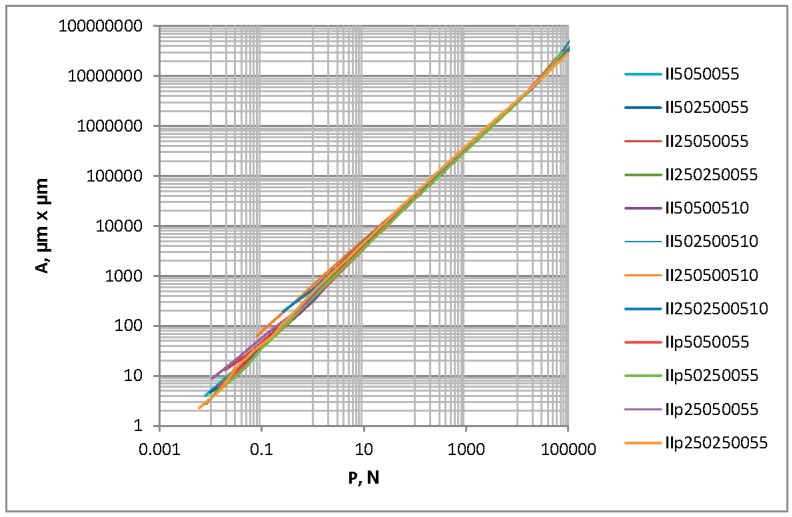
Contact area versus load for random two-process isotropic surfaces, Spq = 0.5 µm.

The similar analysis was carried out for contact of rougher two-process surfaces. The roughness heights of two-process surfaces increased five times. These textures were denoted similarly to the surfaces listed in Table 2. The Spq parameter of all the analyzed surfaces was 0.5 µm; however, the Svq parameter was 5 or 10 µm; meanwhile, the Smq parameter, the correlation lengths of the plateau and valley parts, CLp and CLv, respectively, and the density of the Sds2p summits were the same as those of similar smoother surfaces from Table 2. Due to the amplitude increasing five times, the standard deviation of the surface height σ2ps also increased five times, while the mean radius of the summits’ curvature R2p decreased five times. Due to the surface height increase, plastic contact occurred (the plasticity indices were higher than 1.5). Figure 13 presents the contact area versus load for the contact of these textures.

**Figure 14:**
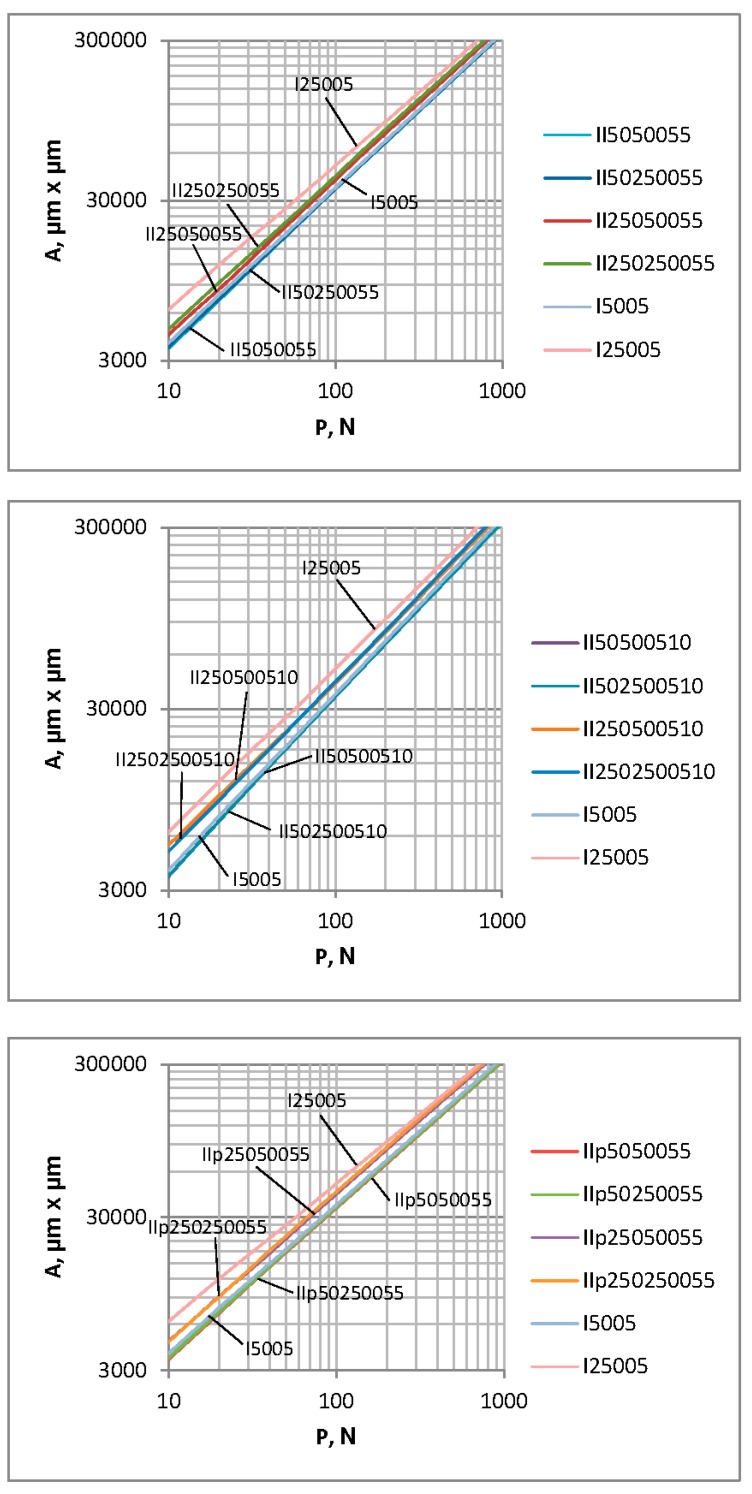
Contact area versus load for random one-process and two-process isotropic surfaces, characterized by the Spq (Sq) parameter of 0.5 µm.

One can see that differences between contact characteristics are visible for smaller loads. In order to better study the deviations between contact characteristics, Figure 14 presents enlarged graphs for three groups of surfaces. For comparison, the contact performances of one-process surfaces I5005 and I25005 (of the Sq parameter 0.5 µm and correlation lengths of 50 and 250 µm, respectively) are also shown.

For various groups of surfaces, the highest contact area was obtained for one-process texture I25005; however, the smallest contact areas were found for one-process texture I5005 and two-process surfaces characterized by the smaller correlation length of the plateau part.

Table 4 presents the values of the contact area for two-process surfaces for loads of 10 and 100 N.

One can see from the analysis of Table 4 that for the load of 10 N, the highest contact areas correspond to surfaces II250500510 and II2502500510; however, the smallest contact areas correspond to surfaces IIp5050055 and II50500510. When the load was larger (100 N), the highest contact area was obtained for surfaces IIp250250055 and II250250055, while the smallest contact area was obtained for topographies IIp5050055 and IIp50250055. The plateau surface part governs the contact characteristics; when the CLp was smaller, the contact area for the given load was also smaller. The effect of the main wavelength of the valley part was smaller than that of the plateau part, but it was also substantial. In the majority of analyzed cases when the CLv was bigger, the contact area for the given load was larger. When the load was 10 N, the ratio between the highest and the smallest contact areas was 1.48, while for the load of 100 N, this ratio decreased to 1.33. For plastic contact, the effect of two-process surface topography on the contact behavior is smaller compared to the elastic contact. One can obtain similar findings after analysis of the contact behavior of one-process textures (see Figure 7).

**Figure 15:**
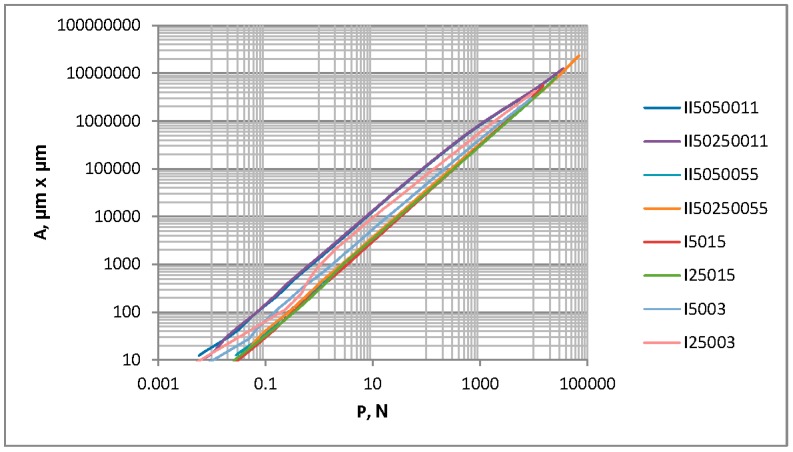
Contact area versus load for random one-process and two-process isotropic surfaces, characterized by Sq parameters of 0.3 and 1.5 µm.

Typically, performances of one- and two-process surfaces are compared for the same standard deviation of surface height Sq [22,23,24]. Two-process surfaces II5050011 and II250250011 are characterized by the Sq parameter of about 0.3 µm; however, surfaces II5050055 and II25025005 are characterized by the Sq parameter of about 1.5 µm. The contact characteristics of surfaces II5050011 and II250250011 were compared with those of one-process textures characterized by the Sq parameter of 0.3 µm and correlation lengths of 50 and 250 µm (I5003 and I25003, respectively). One-process surfaces characterized by the Sq parameter of 1.5 µm and correlation lengths of 50 and 250 µm were denoted as I5015 and I25015, respectively. Their contact behaviors were also taken into consideration. The separation h for the same load or the same contact area was higher for one-process surfaces compared to two-process textures of the same standard deviation of height. This dependence was obtained independently from the reference plane for two-process textures (modal or mean plane). The contact area for the same load was smaller for one-process surfaces compared to two-process textures characterized by the same value of the Sq parameter. This effect was lower for the higher roughness height and probably resulted from the plastic deformation (Figure 15).

**Figure 16:**
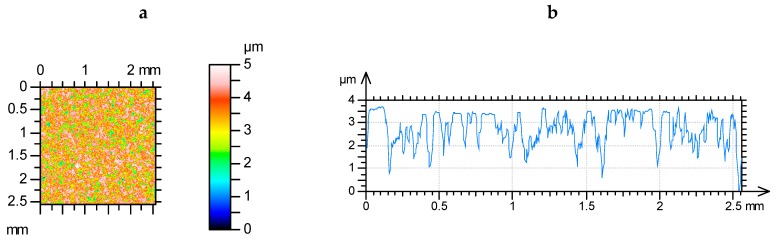

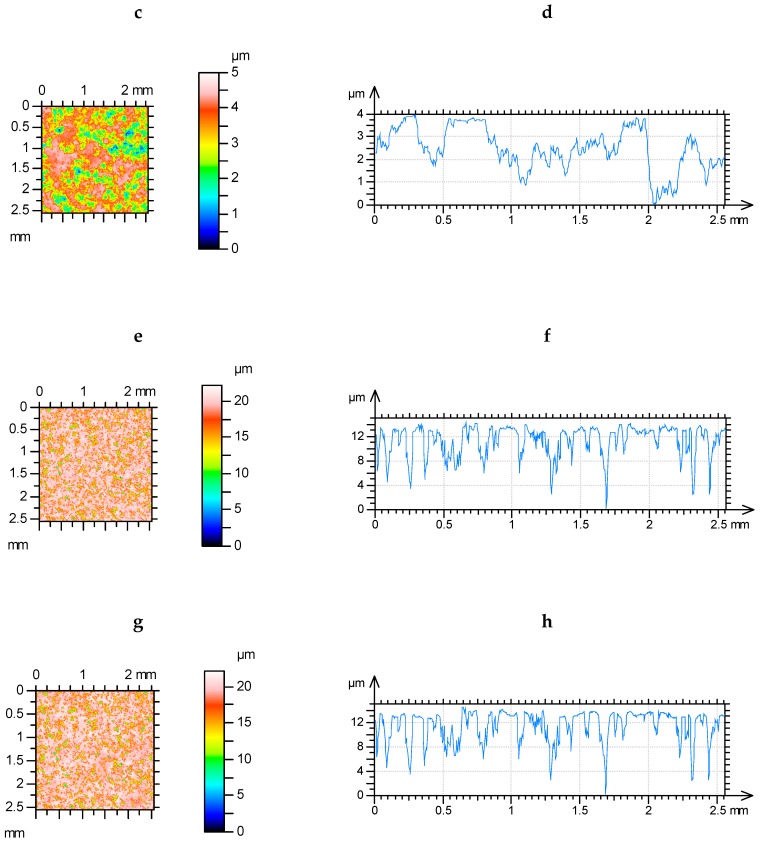
Contour plots (**a**,**c**,**e**,**g**) and profiles (**b**,**d**,**f**,**h**) of two-process surfaces: II25050011 (**a**,**b**), II250250011 (**c**,**d**), IIp5050055 (**e**,**f**), and IIp 25050055 (**g**,**h**).

The contour plots and profiles of selected two-process surfaces are shown in Figure 16. Surfaces II25050011 and II250250011 of various correlation lengths of the valley parts look different. Contrary, topographies IIp5050055 and IIp25050055 of various correlation lengths of the plateau portions look similar. However, the contact of surface IIp25050055 with a smooth flat sample led to a smaller contact area of 27%, compared to surface IIp5050055, when the load was 100 N. For the same load, the change from surface II25050011 to surface II25025001 resulted in a smaller decrease of the contact area of 14%.

## 5. Conclusions

The contact characteristics of a random one-process surface depend on the height and spatial properties. An increase in the main surface wavelength and a decrease in the roughness height cause a decrease in the separation for the given load. The effect of one-process surface texture on the relation between contact area and separation depends on the contact area. The contact area for the same load is higher when the plasticity index is smaller.

The plateau region governs the contact characteristics of a two-process surface; the effect of the valley surface portion is also substantial, but smaller. An increase in the correlation length of the plateau part leads to growth in the contact area for the given load. Typically, an increase in the correlation length of the valley portion also caused an increase in the contact area for the same load. Similar to one-process textures, the contact area is larger for a smaller roughness height.

The plastic deformation leads to a smaller effect of the surface topography on the contact characteristics.

The contact area is smaller for one-process textures compared to two-process topographies of the same standard deviation of the surface height. This effect is lower for higher roughness height.

## Figures and Tables

**Table 1:** Parameters of the analyzed one-process surfaces as well as plasticity indices Ψ.

Surface	CL µm	Sq µm	σ_s_ µm	Sds (µm)^−2^	R µm	Ψ
I5001	50	0.1	0.09	0.00061	333.35	0.43
I10001	100	0.1	0.096	0.00053	479.6	0.37
I25001	250	0.1	0.098	0.00044	773.7	0.3
I5005	50	0.5	0.45	0.00061	66.7	2.15
I10005	100	0.5	0.48	0.00053	95.9	1.85
I25005	250	0.5	0.49	0.00044	154.7	1.5

**Table 2:** Surface topography parameters Spq, Svq, Smq, CLp, and CLv, the standard deviation of summit heights σ2ps, the mean radius of summits curvature R2p and density of summits Sds2p for simulated two-process surfaces. CLp: the correlation length of the plateau surface, CLv: correlation length of the valley surface.

Surface Denotation	Spq, µm	Svq, µm	Smq, %	CLp, µm	CLv, µm	σ2ps, µm	R2p, µm	Sds2p (µm)^−2^
II5050011	0.1	1	84	50	50	0.092	252.1	0.00066
II50250011	0.1	1	84	50	250	0.093	286.7	0.00057
II25050011	0.1	1	84	250	50	0.099	309.2	0.0005
II250250011	0.1	1	84	250	250	0.099	484.2	0.00038
II5050012	0.1	2	84	50	50	0.092	235.2	0.00054
II50250012	0.1	2	84	50	250	0.093	231.6	0.00039
II25050012	0.1	2	84	250	50	0.099	268.1	0.00053
II250250012	0.1	2	84	250	250	0.099	423.3	0.00036
IIp5050011	0.1	1	50	50	50	0.092	178.4	0.00061
IIp50250011	0.1	1	50	50	250	0.093	254.9	0.00046
IIp25050011	0.1	1	50	250	50	0.099	509.1	0.00053
IIp250250011	0.1	1	50	250	250	0.099	619.2	0.00044

**Table 3:** Contact areas for loads of 10 and 100 N for two-process surfaces characterized by the Spq parameter of 0.1 µm.

Surface Denotation	Contact Area [µm^2^] for Load:	
	10 N	100 N
II5050011	12,801	112,546
II50250011	13,242	116,616
II25050011	16,387	140,349
II250250011	18,666	160,697
II5050012	12,802	112,246
II50250012	12,738	106,681
II25050012	19,018	138,032
II250250012	19,022	154,667
IIp5050011	11,945	99,732
IIp50250011	12,936	102,458
IIp25050011	18,436	162,823
IIp250250011	19,474	176,301

**Table 4:** Contact areas for loads of 10 and 100 N for two-process surfaces characterized by the Spq parameter of 0.5 µm.

Surface Denotation	Contact Area [µm^2^] for Load:	
	10 N	100 N
II5050055	3622	35,534
II50250055	3671	35,665
II25050055	4367	42,370
II250250055	4737	42,918
II50500510	3526	35,651
II502500510	3616	34,890
II250500510	5219	40,937
II2502500510	4903	42,312
IIp5050055	3526	33,193
IIp50250055	3626	35,263
IIp25050055	4654	42,249
IIp250250055	4604	44,126
